# Virtual Neuropsychological Assessment After Severe or Critical COVID-19: The NeuroCov Pilot Feasibility Study

**DOI:** 10.7759/cureus.106960

**Published:** 2026-04-13

**Authors:** Kimia Honarmand, Sydni G Paleczny, Krista Wright, John Basmaji, Michelle Y Wong, Marat Slessarev, Cheryl Forchuk, Karen J Bosma, Adrian M Owen

**Affiliations:** 1 Division of Critical Care, Department of Medicine, McMaster University, Hamilton, CAN; 2 Department of Neuroscience, Schulich School of Medicine and Dentistry, Western University, London, CAN; 3 Division of Critical Care, Department of Medicine, London Health Sciences Centre, London, CAN; 4 Division of Critical Care, Department of Medicine, Western University, London, CAN; 5 Arthur Labatt Family School of Nursing and Department of Psychiatry, Western University, London, CAN; 6 Department of Physiology and Pharmacology, Western University, London, CAN

**Keywords:** cognitive function, cognitive neuroscience, covid-19, critical care and hospital medicine, critically ill patients, digital cognitive assessment, mental health, neuropsychological assessment

## Abstract

Purpose

Survivors of COVID-19 experience a myriad of psychological and cognitive complications that persist beyond the period of acute illness. Neuropsychological assessment has traditionally involved in-person testing, which may be inconvenient and costly. We evaluated the feasibility of a remote/virtual assessment tool to characterize neuropsychological outcomes among survivors of severe or critical COVID-19 illness.

Methods

We conducted a pilot feasibility study evaluating neuropsychological outcomes of a convenience sample of adult patients who survived severe/critical COVID-19 (the “NeuroCov" study) at six and 12 months after discharge from one of two participating hospital sites in London, Canada. Primary feasibility outcomes included recruitment and completion rates, as well as reported technological issues. Secondary outcomes included neuropsychological and functional assessment. Patients completed the web-based Creyos neurocognitive battery (Creyos, Toronto, Canada), questionnaires for depression, anxiety, and post-traumatic stress, and a telephone interview to assess functional status. Patients remotely self-administered the assessments using a computer or tablet device.

Results

We enrolled 28 patients six and/or 12 months after hospitalization for severe or critical COVID-19 (recruitment rate of 14.3%). Technological challenges with the remote testing platform were rare. Consent rates were 63% for the six-month and 61% for the 12-month follow-up. Nineteen of 28 patients (68%) had at least mild impairment on two or more of the six Creyos tests administered, primarily on verbal processing; 25 (89%) had at least mild depression, 19 (68%) had at least mild generalized anxiety, and seven (25%) reported symptoms of probable post-traumatic stress. Patients reported persistent fatigue, pain/discomfort, and problems with mobility after hospital discharge.

Conclusion

A virtual neuropsychological assessment platform is feasible to self-administer by patients after hospital discharge, but data were limited by somewhat low participation rates. Future research should examine the accuracy and reliability of the Creyos tool in hospital settings so that the virtual platform may be used in future clinical research evaluating various therapeutic interventions.

## Introduction

Patients who survive COVID-19, particularly those with severe or critical illness, experience a myriad of long-term complications, including neuropsychological symptoms [1-6]. These neuropsychological effects are often colloquially referred to as “brain fog” [4] and include deficits such as short-term memory and reduced concentration, persisting long beyond the period of acute illness [5].

Studies using formal neuropsychological testing indicate that cognitive impairment affects approximately 26-67% of COVID-19 survivors [3-5], with substantial variability depending on illness severity and timing of assessment [6]. Psychiatric morbidity is also frequently observed, with up to half of individuals reporting symptoms of depression or anxiety in the post-COVID period [6].

Despite increasing recognition of post-COVID-19 neuropsychological sequelae, access to formal neuropsychological assessment is limited. Traditional neuropsychological evaluation entails in-person testing by a trained neuropsychologist in a hospital or clinic. In-person neuropsychological assessment may be inconvenient for patients, time-consuming for practitioners, impractical due to infection control practices during a pandemic, and overall costly for the healthcare system. This limits the proportion of patients, particularly those in geographically remote areas, who can be evaluated. Further, most studies evaluating the long-term neuropsychological sequelae of COVID-19 have been implemented in outpatient settings, which may disproportionately disadvantage patients with geographical, mobility, or financial limitations prohibiting in-person assessment, that can bias study findings [7,8].

Remote or virtual neuropsychological assessment has emerged as a potential solution to these limitations. Advancements in web-based neurocognitive assessment now allow patients to self-administer cognitive tasks using personal devices, enabling scalable assessment across geographically dispersed populations. Prior studies in ICU survivors [9] and other patient populations [10,11] have demonstrated that web-based cognitive batteries can detect clinically meaningful cognitive impairment. However, data on the feasibility of such platforms among survivors of severe COVID-19 beyond the early post-discharge period remain limited.

Accordingly, the objectives of the “NeuroCov" pilot study were twofold: first, to evaluate the feasibility of remote, self-administered neuropsychological assessment using a web-based cognitive battery among survivors of severe or critical COVID-19 at six and 12 months following hospital discharge; and second, to characterize the cognitive, psychological, and functional status of patients as hypothesis-generating outcomes in this cohort.

## Materials and methods

Project design

We conducted a pilot prospective observational study to evaluate neuropsychological outcomes of patients at six and 12 months (±2 months) after hospital discharge following recovery from severe or critical COVID-19. Appendix 1 presents the STrengthening the Reporting of OBservational studies in Epidemiology (STROBE) checklist. The study was approved by Western University’s Research Ethics Board (#116575; approved April 08, 2021), and all patients provided electronic informed consent.

Patient eligibility criteria

We recruited a convenience sample of adult patients (aged 18 years or older) from one of two participating tertiary care hospitals in London, Canada (University Hospital and Victoria Hospital). Patients were eligible to participate if they had a confirmed diagnosis of COVID-19 between March 2020 and April 2022, survived hospital discharge after being admitted to either of the following settings: (1) intensive care unit (ICU) at either hospital or (2) a non-ICU setting with severe disease, and were co-managed by the ICU outreach team. We excluded patients with pre-existing dementia, stroke, traumatic brain injury, visual or motor impairment that prohibits the use of a computer/tablet device, inability to read or comprehend English, and no access to a computer or tablet device with an internet connection.

Demographic and clinical data

We recorded demographic and clinical data that may influence long-term neurological outcomes (Appendix 2) via electronic medical records and telephone interviews with each patient.

Study procedures

We reviewed a database of patients admitted to the hospital with COVID-19 between March 2020 and April 2022. We reviewed patients’ electronic charts to assess for eligibility. Then, the study team contacted potentially eligible patients by telephone to confirm eligibility, discuss the study, and invite them to participate. Patients performed all study tasks remotely using a computer or tablet device. We provided a link to the study REDCap platform (Vanderbilt University, Nashville, TN) via email, where the participant reviewed and electronically signed the informed consent form and completed study assessments. We also performed a telephone interview to determine patients’ symptoms, functional status, and self-reported healthcare utilization after hospital discharge.

Feasibility outcomes

We evaluated study feasibility by recording the consent rate (proportion of eligible patients who consented to participate in the study) and follow-up rate (proportion of patients who completed follow-up assessments at each time point). To determine the feasibility of self-administration of the virtual neuropsychological battery, we recorded challenges related to the remote nature of study procedures and the technological platform (i.e., online questionnaires and the Creyos-6 battery (Creyos, Toronto, Canada)).

Neuropsychological outcomes

We evaluated cognitive function using the Creyos cognitive battery (previously called Cambridge Brain Sciences (CBS); www.creyos.com); permission to use was obtained via an established contract outlining platform use terms and subscription conditions [12]. The Creyos battery has been administered in several large patient cohorts [12,13] and has a large normative database (> 85,000) that can be used to conduct age- and sex-matched comparisons with patient performance. The normative database is designed to be representative of the general population, making it robust for valid comparisons with the demographic profile of the present cohort. Our team previously showed that the use of Creyos is feasible for use in ICU survivors and that the six-test version performs similarly to the 12-test version in detecting cognitive impairment [9]. We assessed cognition using the six-test version of Creyos (Creyos-6, previously called the CBS-6 [9]).

We evaluated the presence and severity of psychological symptoms using open-access, validated questionnaires that have been widely used in previous research to evaluate psychological outcomes among critical illness survivors: (1) Nine-Item Patient Health Questionnaire (PHQ-9 [14]) to measure depressive symptoms, (2) Seven-Item Generalized Anxiety Disorder (GAD-7 [15]) scale to measure anxiety symptoms, (3) 22-item Impact of Events Scale Revised (IES-R [16]) for post-traumatic stress symptoms.

Functional status and healthcare utilization

We administered the open-access COVID-19 Yorkshire Rehabilitation Screening (C19-YRS) Questionnaire via a telephone interview with each patient. The C19-YRS [17] evaluates patients’ symptom profile, functional status, and healthcare utilization before and after contracting COVID-19.

Data analysis

We reported descriptive statistics (means and standard deviations, medians and interquartile ranges, and proportions and percentages, as appropriate) to summarize demographic and clinical characteristics. We reported scores on the psychological instruments and classified psychological symptoms in severity based on established cut-off scores (Appendix 3). We defined cognitive impairment as a score ≥1.5 standard deviations below the score of age- and sex-matched controls on each Creyos test and mild cognitive impairment as a score of ≥ 1.0 standard deviations below the score of age- and sex-matched controls. We calculated patients’ performance on each of three cognitive domains (short-term memory, reasoning, and verbal skills) using factor loading [13], a process where the z-score on an individual test is multiplied by a value reflective of the weight of a particular domain in that test [13]. We assessed data distribution for normality, and appropriate parametric or non-parametric tests were applied accordingly. For longitudinal outcomes, analyses at each time point were conducted using available data (complete-case analysis). Participants who did not complete the 12-month follow-up were excluded from 12-month analyses. The number of participants included at each stage and reasons for attrition are presented in the first figure under Results section.

## Results

Patient characteristics

We enrolled 28 patients (11 women; median age 63, IQR 48.75 to 70.25). Table 1 summarizes patients’ demographics, and Table 2 summarizes their clinical characteristics. Twenty-one patients were admitted to the ICU for a median of seven days (IQR 5 to 21; range 2 to 50), and 14 patients received invasive mechanical ventilation for a median of seven days (IQR 6 to 18; range 1 to 31). Median length of hospital stay was 17 (IQR 12 to 39) for patients admitted to the ICU during their hospitalization and 12 (IQR 9 to 19) for patients who were admitted to a non-ICU setting and followed by the critical care outreach team due to severe illness.

**Table 1:** Patients’ Demographic and Clinical Characteristics

Characteristics	All patients	Patients admitted to ICU (critical COVID-19) (n = 21)	Patients followed by ICU outreach team on the general ward (severe COVID-19) (n = 7)
Age, median (IQR)	61.5 (52-69)	60 (49-67)	64 (54.5-71.5)
Sex, n (%)			
Female	11 (34.4)	8 (32.0)	3 (42.9)
Male	21 (65.6)	17 (68.0)	4 (47.1)
Marital status, n (%)			
Married or common-law relationship	14 (66.7)	10 (62.5)	4 (80.0)
Not married	7 (33.3)	6 (37.5)	1 (20.0)
Highest level of education, n (%)			
High school degree or equivalent	5 (23.8)	4 (25.0)	1 (20.0)
Vocational or technical school	1 (4.8)	1 (6.2)	0 (0)
Some college, no degree	1 (4.8)	1 (6.2)	0 (0)
College or associate degree	6 (28.5)	3 (18.8)	3 (60.0)
Bachelor’s degree	8 (38.1)	7 (43.8)	1 (20.0)
Occupation, n (%) *based on #18 on C19*			
Change in employment status compared to pre-COVID			
Yes	9 (42.9)	8 (50.0)	1 (20.0)
No	12 (57.1)	8 (50.0)	4 (80.0)
How has your work status changed since your hospitalization with COVID-19?			
No change	12 (57.1)	8 (50.0)	4 (80.0)
Reduced or modified work hours/duties	3 (14.3)	2 (12.5)	1 (20.0)
Not working outside the home	4 (19.0)	4 (25.0)	0 (0.0)
Other	2 (9.5)	2 (12.5)	0 (0.0)
Substance use prior to illness, n (%)			
Smoking	9 (42.9)	7 (43.8)	2 (40.0)
Vaping	0 (0.0)	0 (0.0)	0 (0.0)
Alcohol	12 (57.1)	9 (56.3)	3 (60.0)
Cannabis	2 (9.5)	0 (0.0)	2 (40.0)
Recreational drugs	0 (0.0)	0 (0.0)	0 (0.0)
None	6 (28.6)	4 (25.0)	2 (40.0)
Pre-admission comorbidities, n (%)			
Coronary artery disease	3 (10.7)	3 (18.8)	0 (0.0)
Heart failure	4 (14.3)	3 (18.8)	1 (20.0)
Previous cardiac arrest	2 (7.2)	2 (12.5)	0 (0.0)
Atrial fibrillation	2 (7.2)	2 (12.5)	0 (0.0)
Hypertension	11 (39.3)	9 (56.3)	2 (40.0)
Diabetes	5 (17.9)	4 (25.0)	1 (20.0)
Respiratory illness	3 (10.7)	1 (6.3)	2 (40.0)
Dyslipidemia	8 (28.5)	7 (43.8)	1 (20.0)
Liver disease	1 (3.6)	1 (6.3)	0 (0.0)
Kidney disease	1 (3.6)	1 (6.3)	0 (0.0)
Depression	5 (17.9)	4 (25.0)	1 (20.0)
Anxiety	2 (7.2)	1 (6.3)	1 (20.0)

**Table 2:** Patients’ Clinical Course in Hospital

Characteristics	All patients	Patients admitted to ICU (n = 21)	Patients followed by ICU outreach team on the general ward (n = 7)
Highest level of respiratory support, n (%)			
Invasive mechanical ventilation	14 (50.0)	13 (61.9)	1 (14.2)
Non-invasive mechanical ventilation	7 (25.0)	6 (28.5)	1 (14.2)
High flow nasal cannula	3 (10.7)	1 (4.8)	2 (28.6)
Oxygen therapy only	3 (10.7)	1 (4.8)	2 (28.6)
No oxygen therapy	1 (3.6)	0 (0.0)	1 (14.2)
ICU length of stay (days), median (IQR)	7.0 (5-20.8)	7.0 (5-20.8)	NA
Hospital length of stay (days), median (IQR)	16 (11.8-39)	19.5 (13.8 - 39)	12 (8.5-19)
Duration of vasopressor therapy (days), median (IQR)	5.5	6	2
Renal replacement therapy			
Yes	2 (7.1)	2 (9.5)	0 (0)
No	26 (92.9)	19 (90.5)	7 (100)
Duration of hemodialysis, median (IQR)	7.5 (4.3-10.8)	7.5 (4.3-10.8)	0 (0)

Feasibility outcomes

**Figure 1:**
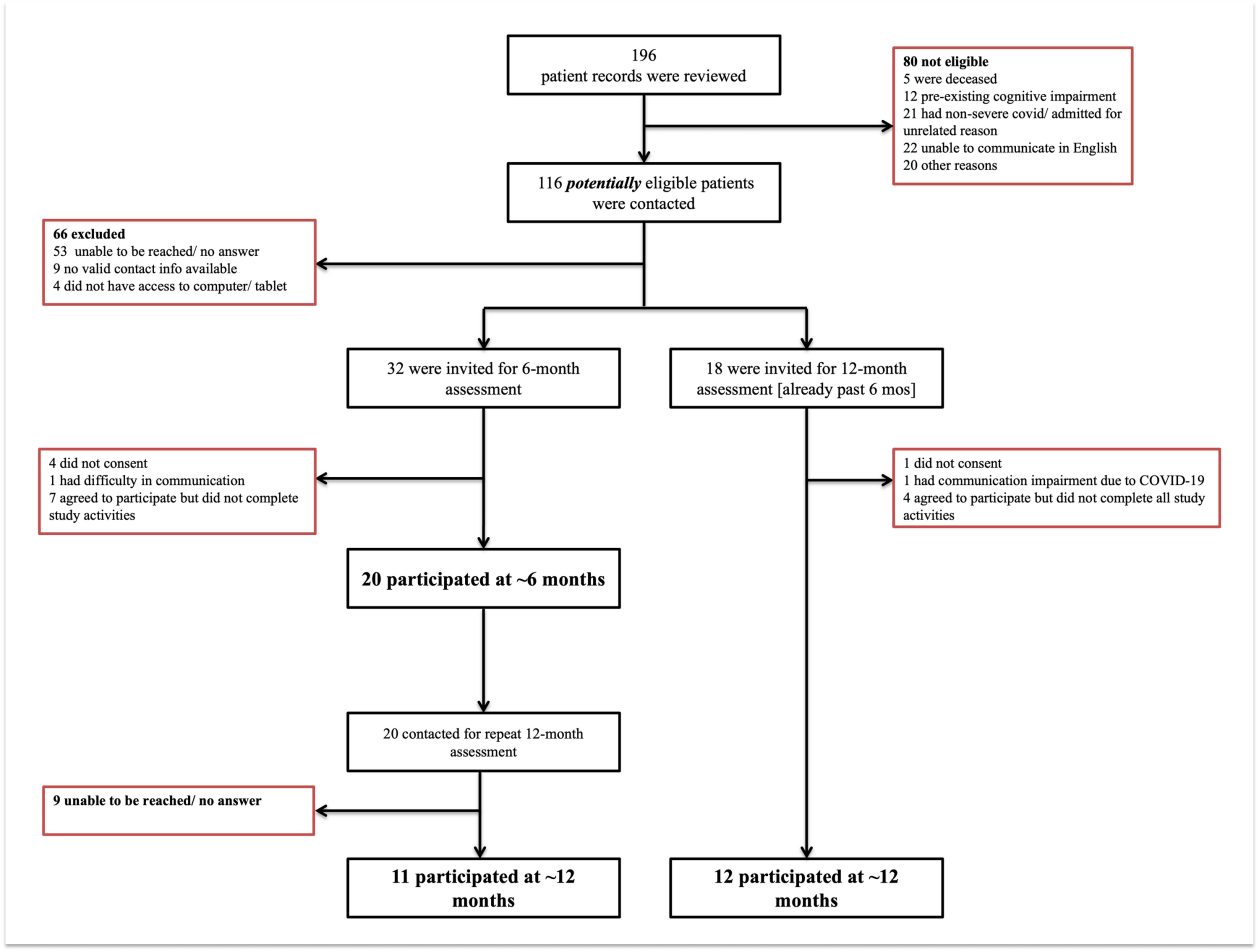
Recruitment Flow Diagram Flow diagram of all participants recruited to the study. Of 32 total participants, four patients did not complete the Creyos battery at any time and were therefore not included in subsequent analyses. Image was created using Microsoft PowerPoint (Microsoft® Corp., Redmond, WA) and GraphPad Prism (version 9.4.1) (GraphPad Software, San Diego, CA).

Figure 1 summarizes study recruitment and participation at each stage. Among 196 patients reviewed, 50 were invited to participate in the study (32 for the six-month assessment and 18 for the 12-month assessment). Twenty of 32 patients provided electronic consent to participate at six-month follow-up, yielding a 63% consent rate at six-month follow-up (Figure 1). Among 38 patients who were invited to participate in the 12-month assessment, 23 provided electronic consent to participate at 12-months, yielding a follow-up rate of 61% at 12-month follow-up. Among consenting participants, four did not complete the Creyos battery, yielding 28 total patients included in the analyses. In terms of the feasibility of the remote testing platform, participants experienced a few technological challenges (i.e., misplaced account information), which were resolved through email or telephone instructions provided by the study team.

Neurological complications during hospitalization

Among seven patients who had brain imaging (head CT: n = 6; head CT and brain MRI: n = 1) during the index hospitalization, one patient had an acute intraparenchymal hemorrhage within the right basal ganglia and corona radiata and possible post-infectious hemorrhagic leukoencephalitis; the remaining six patients had no acute findings on brain imaging. One of 28 patients had seizure activity during hospitalization, diagnosed based on clinical findings. One other patient was clinically diagnosed with “encephalopathy” during hospitalization based on an EEG.

Neuropsychological outcomes after hospital discharge

Neuroimaging Findings

Among two patients who had head CT (n = 1) and brain MRI (n = 1) following discharge from the hospital, neither showed any acute abnormalities.

Psychiatric outcomes

Among 22 patients who participated in the C19-YRS telephone questionnaire, eight reported new or worsened depressive symptoms (Appendix 4). On the PHQ-9, 25 of 28 patients (89.3%) reported at least mild symptoms of depression on the PHQ-9: 12 of 28 (42.8%) mild, 5 (17.9%) moderate, 6 (21.4%) moderately severe, and 2 (7.1%) severe depressive symptoms. The most frequently reported symptoms of depression were being tired and fatigued, followed by trouble falling asleep or staying asleep or oversleeping, and changes in appetite. Five (17.9%) patients reported thoughts of suicide since the index hospitalization on the PHQ-9 (Table 3).

**Table 3:** Patients’ Psychological Outcomes

Domain	All patients (n = 28)	Six months (n = 17)	12 months (n = 18)
Symptoms of depression			
Total PHQ-9 score, median (IQR)	9 (6-15.25)	8 (6-14)	10.5 (8-16)
PHQ-9 depressive symptom severity, n/total N (%)			
Minimal depression 0-4	3 (10.7)	2 (11.8)	1 (5.5)
Mild depression 5-9	12 (42.8)	8 (47.1)	8 (44.4)
Moderate depression 10-14	5 (17.8)	3 (17.6)	3 (16.7)
Moderately severe depression 15-19	6 (21.4)	3 (17.6)	3 (16.7)
Severe depression 20-27	2 (7.1)	1 (5.9)	3 (16.7)
Symptoms of generalized anxiety			
Total GAD-7 score, Median (IQR)	6 (3.75-9)	5 (3-7)	7.5 (4.3-11)
GAD-7 anxiety symptom severity			
Minimal anxiety 0-4	9 (32.4)	6 (35.3)	5 (27.8)
Mild anxiety 5-9	13 (46.4)	10 (58.8)	5 (27.8)
Moderate anxiety 10-14	5 (17.8)	1 (5.9)	7 (38.9)
Severe anxiety 15-21	1 (3.8)	0 (0.0)	1 (5.5)
Symptoms of post-traumatic stress			
Total IES-R score, median (IQR)	17 (10-33)	16 (10-28)	19 (7.8-41.5)
IES-R post-traumatic stress severity, n/total N (%)			
IES-R not a clinical concern	17 (60.7)	11 (64.7)	10 (55.5)
Clinical concern for PTSD	11 (39.3)	6 (35.3)	8 (44.5)
IES-R subscale scores, mean (SD)			
Intrusion subscale (The mean response of items 1, 2, 3, 6, 9, 14, 16, 20)	1.03 (0.77)	0.92 (0.60)	0.94 (0.84)
Avoidance subscale (The mean response of items 5, 7, 8, 11, 12, 13, 17, 22)	0.93 (0.93)	0.95 (0.99)	1.19 (0.89)
Hyperarousal subscale (The mean response of items 4, 10, 15, 18, 19, 21)	0.84 (0.95)	0.65 (0.70)	1.05 (1.07)

Ten of 22 patients who participated in the C19-YRS telephone questionnaire reported new or worsened anxiety symptoms (Appendix 4). On the GAD-7, 19 of 28 patients (67.8%) had at least mild symptoms of generalized anxiety on the GAD-7: 13 of 28 (46.4%) mild, 5 (17.9%) moderate, and 1 (3.6%) severe anxiety symptoms. The most frequently reported symptoms of anxiety were worrying about different things, followed by being easily annoyed or irritable, and trouble relaxing (Table 3).

Six of 22 patients who participated in the C19-YRS telephone questionnaire reported unwanted memories of their illness/ hospitalization, seven reported unpleasant dreams about their illness/hospitalization, and 12 reported trying to avoid thoughts or feelings about their illness/hospitalization (Appendix 4). On the IES-R, seven of 28 patients (25.0%) had an IES-R score ≥ 33, which indicates a probable diagnosis of PTSD (Table 3). Among the subscales of the IES-R, patients scored highest on the intrusion subscale, which indicates that frequent mental re-experiences of their period of illness/hospitalization are the primary concern.

Neurocognitive outcomes

Thirteen of 22 patients who participated in the C19-YRS questionnaire reported new problems with concentration, 15 reported new problems with memory, and 9 reported changes in the way they communicate as noticed by others (Appendix 4).

**Figure 2:**
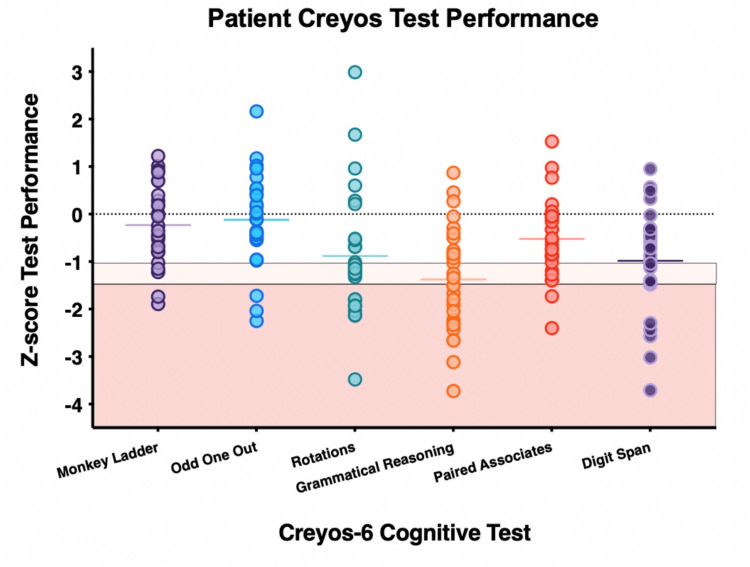
Patients’ Cognitive Performance on Individual Tests of the Creyos-6 Battery Patient performance indicated by Z-scores compared to age- and sex-matched healthy controls on each of 6 Creyos cognitive tests. Individuals scoring <1.5 SDs evidence marked impairment (red shaded box); those scoring <1.0 SD of the mean evidence mild impairment (pink shaded box) (N = 28). Note: Merged follow-up from 28 patients between six and 12 months post-discharge. Data from the 12-month follow-up are presented from patients who completed the Creyos battery at both six and 12 months. Six-month data from 10 patients, 12-month data from 18 patients. Image was created using Microsoft PowerPoint (Microsoft® Corp., Redmond, WA) and GraphPad Prism (version 9.4.1) (GraphPad Software, San Diego, CA).

Eighteen of 28 patients who completed the Creyos-6 cognitive battery (64.3%) demonstrated marked impairment (defined as a score ≥1.5 standard deviations below the score of age- and sex-matched controls on any Creyos test) on at least one cognitive test. Nineteen patients (67.8%) demonstrated at least mild impairment (defined as a score ≥1.0 standard deviations below the score of age- and sex-matched controls on any Creyos test) on at least two of the tests they completed. Only 5 patients (17.9%) did not show impaired performance on any of the Creyos tasks. Appendix 5 shows the average Z-score for the primary measure of each of the 6 Creyos tests, and Figure 2 displays the individual participant performances on the Creyos battery tests.

**Figure 3:**
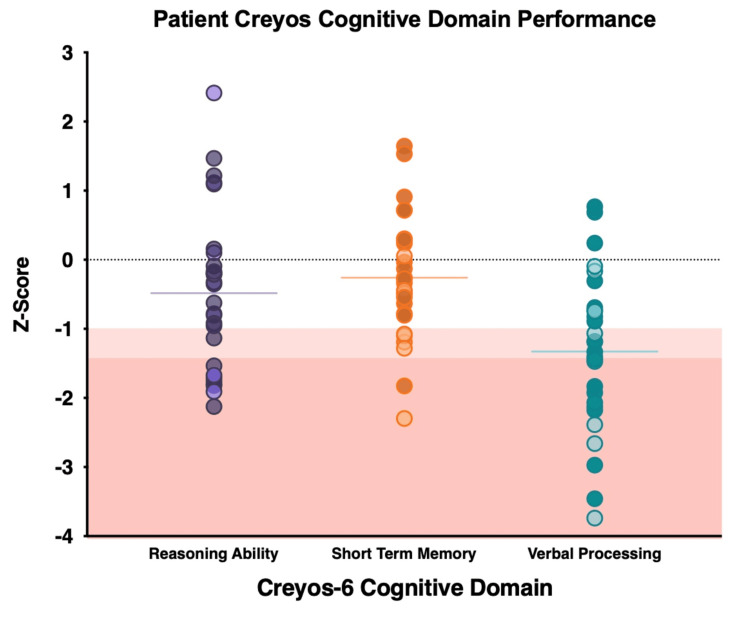
Patient Performance on Cognitive Domains Patient performance on each of three cognitive domains (reasoning ability, short-term memory, and verbal processing) was indicated by Z-scores compared to age- and sex-matched healthy controls. Individuals scoring <1.5 SDs evidence marked impairment (red shaded box); those scoring <1.0 SD of the mean evidence mild impairment (pink shaded box) (N = 28). Note: Darker colors indicate 12-month data. Merged follow-up from 28 patients between six and 12 months post-discharge. Data from the 12-month follow-up are presented from patients who completed the Creyos battery at both six and 12 months. Six-month data from 10 patients, 12-month data from 18 patients. Image was created using Microsoft PowerPoint (Microsoft® Corp., Redmond, WA) and GraphPad Prism (version 9.4.1) (GraphPad Software, San Diego, CA).

Performance on each of three cognitive domains (reasoning, verbal skills, short-term memory) was calculated using factor loading, as demonstrated in previous studies [13]. Among 28 patients who completed the Creyos-6 battery, eight patients (29%) showed at least mild impairment on short-term memory, nine (32%) on reasoning ability, and 17 patients (60.7%) showed at least mild impairment on verbal processing skills. Ten patients (36%) showed impairment in at least two out of three cognitive domains. Figure 3 displays patients’ cognitive performance on each of the three cognitive domains evaluated by the Creyos cognitive battery.

**Figure 4:**
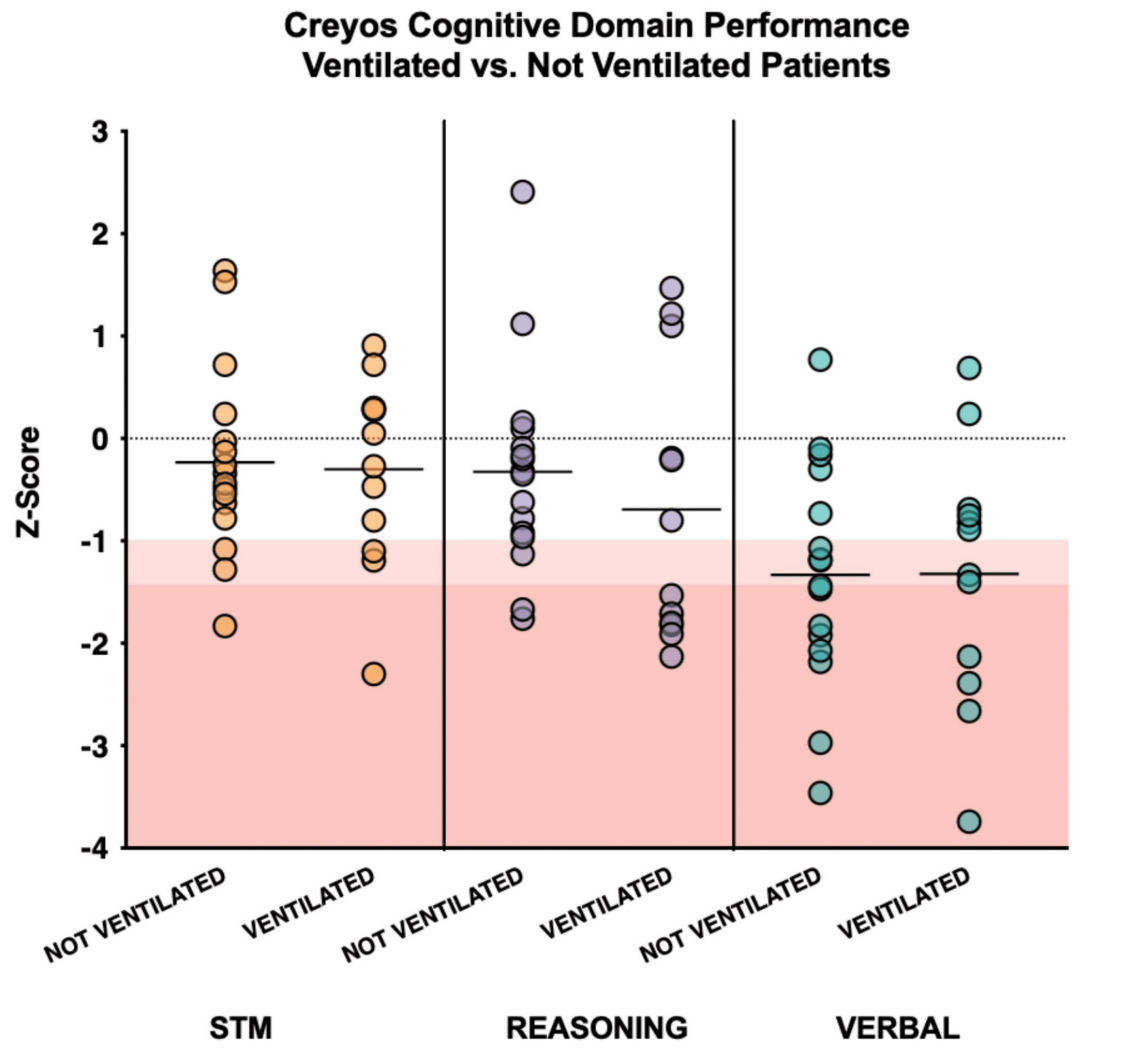
Patient Performance on Cognitive Domains by Whether or Not They Received Invasive Mechanical Ventilation Patient performance on three cognitive domains (reasoning ability, short-term memory, and verbal skills), indicated by Z-scores compared to age- and sex-matched healthy controls by whether or not they received invasive mechanical ventilation. Individuals scoring <1.5 SDs evidence marked impairment (red shaded box); those scoring <1.0 SD of the mean evidence mild impairment (pink shaded box). Note: Merged follow-up from 28 patients between six and 12 months post-discharge. Data from the 12-month follow-up are presented from patients who completed the Creyos battery at both six and 12 months. N = 12 ventilated, N = 16 not ventilated. Image was created using Microsoft PowerPoint (Microsoft® Corp., Redmond, WA) and GraphPad Prism (version 9.4.1) (GraphPad Software, San Diego, CA).

We evaluated the cognitive performance of patients who received mechanical ventilation compared to those who did not receive invasive ventilation. Although the relatively small sample size precludes statistical comparison, visual inspection of the data indicates that patients who received invasive ventilation (n = 12; mean z-score -0.69, SD 1.34) had lower reasoning ability, reflecting poorer executive functioning, compared to those who did not receive invasive ventilation (n = 16; mean z-score -0.32, SD 1.02) (Appendix 6; Figure 4).

Persistent physical symptoms and functional status at follow-up

Appendix 4 presents the results of the C19-YRS questionnaires administered to 22 patients (15 at six months, 10 at 12 months). Patients reported new or worsened breathlessness at rest (n = 8), while dressing themselves (n = 10), and while walking up a flight of stairs (n = 16).

The most frequently reported symptoms included fatigue, pain/discomfort, and problems with mobility, reported by 18, 15, and 14 patients, respectively. Seven patients reported new or worsened sleep problems. Eleven patients reported new laryngeal/ airway symptoms (e.g., troublesome coughing or noisy breathing), six reported new swallowing difficulties, and 14 reported new changes in their voice (e.g., difficulty being heard, altered quality of voice, or tiring of voice by the end of the day). Five patients reported new problems with nutrition (e.g., weight loss). Patients also endorsed new problems with bladder (n = 7) and bowel (n = 4) continence.

Seventeen patients reported new or worsened difficulties performing their usual daily activities, and eight reported new or worsened difficulties with personal care (e.g., dressing or washing themselves). Sixteen patients reported worsened global health status, nine reported that they need more assistance with instrumental activities of daily living, and seven reported that their family members have noticed changes or deterioration in the patient’s health. Fifteen patients were working part-time or full-time prior to their illness. Among these, six were no longer working, and two were on reduced/modified work.

Self-reported healthcare utilization

Seven of 22 patients who completed the C19-YRS questionnaire presented to the hospital again at least once following index hospitalization. Reasons included cardiac or respiratory symptoms (n = 3), neurological symptoms (n = 2), follow-up brain imaging (n = 1), pre-scheduled outpatient appointment (n = 1), and reason presumed to be unrelated to history of COVID-19 (i.e., shoulder surgery; n = 1). Four patients required readmission to the hospital since the index hospitalization, three of whom had presented with cardiac or respiratory symptoms. Eight of 21 patients were referred to specialist services (including cardiac, respiratory, and other specialists), and two were followed by the COVID-19 follow-up clinic.

## Discussion

In this pilot feasibility study, we used a web-based platform for remote neuropsychological testing among survivors of severe/critical COVID-19. We found that this remote approach was limited by low patient recruitment, but the use of the web-based platform itself was feasible. We also found neurocognitive impairment and psychiatric disturbances among COVID-19 survivors. The most affected cognitive domain was verbal processing ability, which may indicate a domain-specific pattern of dysfunction. Patients who required invasive mechanical ventilation had lower reasoning skills on visual inspection of the data, indicating more executive dysfunction. However, these findings are limited by a relatively small sample size, and larger prospective longitudinal studies are needed to validate these findings using the proposed virtual/remote assessment tools utilized in this study.

Previous studies using traditional in-person neuropsychological testing have shown that psychological morbidity is common after COVID-19: 13 to 31% of patients experience depressive symptoms [18-20], 18 to 34% report symptoms of anxiety [18,19], and 15 to 33% have symptoms of post-traumatic stress [18-20]. Persistent mood symptoms are more prevalent among those with higher disease severity [18]. Studies of patients who have recovered from COVID-19 have also reported a high incidence of cognitive impairment [7-11], particularly among those who survived severe or critical COVID-19 [11,21].

Survivors of critical illness from other etiologies suffer from neuropsychological sequelae long after recovering from acute illness. One year after hospital discharge, one-third of patients have symptoms of anxiety, one-third have symptoms of depression, and up to half have post-traumatic stress symptoms [22]. The higher rates of psychological symptoms and cognitive impairment in our cohort likely reflect differences in case-mix, as our cohort consisted exclusively of patients with severe/critical illness, whereas prior studies often included broader ICU or hospitalized populations. Among critically ill patients in particular, studies have demonstrated that at the time of hospital discharge, all patients are cognitively impaired [9] and 25 to 80% have cognitive impairment that persists for several years [23], with links to decreased quality of life [24].

The mechanisms that lead to long-term complications in ICU survivors are multifactorial, including the pathophysiology of the illness (e.g., hypoxia may lead to cognitive impairment of variable severity) [25], underlying etiology (i.e., higher incidence of cognitive impairment among patients with acute respiratory distress syndrome) [23], and ICU therapies (e.g., sedatives or corticosteroids) [26].

This study also showed persistence of diminished functional status and modified employment status six to 12 months after hospitalization with severe/critical COVID-19. A similar study using the Creyos battery to measure cognitive function in participants who self-reported having COVID-19 found a similar pattern of results and demonstrated that the cognitive profile of dysfunction may be related to the physical well-being of COVID-19 survivors and less related to mental health symptoms [2]. Together, these findings point to a need for more universal availability of post-COVID-19 recovery clinics to provide patient support, promote patient recovery, and potentially curtail healthcare utilization.

The study was limited by a low recruitment rate, but adequate feasibility in the use of the virtual platform itself. Similarly, a previous pilot study of cognitive outcomes in ICU survivors demonstrated that most patients accessed the link to the Creyos battery and navigated through the tests with few difficulties [9]. Recruitment rate in the present study (63% at six months and 61% at 12 months) was driven by patients' low motivation to partake in the research study, rather than by pragmatic or technological challenges with the virtual platform. Of note, previous studies requiring in-person participation have demonstrated lower recruitment rates, ranging from 26 to 52% [27,28]. As the remote assessment nature of the present study requires participants to have a necessary device and internet connection, it is possible that this design may disproportionately exclude lower socioeconomic groups. In addition, while the Creyos battery compares patient data with a large age- and sex-matched normative database, the lack of individual pre-illness baseline assessment makes it difficult to definitively attribute all cognitive impairment directly to COVID-19; nonetheless, COVID-19 is likely a major contributor. Given the exploratory nature of this study, we did not perform a formal a priori sample size or adjustment for multiple comparisons; as such, results should be interpreted as exploratory.

We postulate several strategies to improve the study recruitment rate. One approach is to shorten the length of the neuropsychological assessment (i.e., fewer questionnaires). In addition, in this study, the cognitive assessments and questionnaires were on separate platforms, requiring patients to switch between web browsers to complete both components. Merging the platforms into a single continuous process may further facilitate full participation in future studies. In addition, providing patients with incentives may increase research participation. This may include non-monetary incentives such as offering follow-up consultations with a clinician to review their cognitive test results and propose cognitive strategies to mitigate their impacts. Indeed, prior studies have shown that monetary [29] and non-monetary [30] incentives improve participation in clinical research.

## Conclusions

Characterizing the scope of neuropsychological impairment after ICU survival will enable implementation of optimal supports and inform research into preventative (i.e., optimizing inpatient care to protect neurological function) and treatment (e.g., cognitive rehabilitation) measures. Traditional neuropsychological testing often involves in-person testing by a trained neuropsychologist, which can be inconvenient for patients (necessitating a return to the clinic for assessment) and time-consuming and costly for the healthcare system. Remote approaches to neuropsychological testing may mitigate these barriers by offering a feasible approach to identify at-risk patients and may facilitate larger-scale prospective studies. Our study establishes a virtual approach that can be used to track the natural history of long-term neuropsychological impairment that may address these barriers.

We found high feasibility in the use of the remote platform for assessing neuropsychological function remotely, but the study was limited by a low recruitment and protocol completion rate. This platform may be used in future clinical research to validate findings in large-scale cohorts and to evaluate various therapeutic interventions without notable platform or technological challenges. Future studies may evaluate strategies to improve study feasibility.

## Appendices

Appendix 1

**Table 4:** STrengthening the Reporting of OBservational studies in Epidemiology (STROBE) Checklist STROBE Statement - checklist of items that should be included in reports of observational studies.

	Item no.	Recommendation	Page no.	Relevant text from manuscript
Title and abstract	1	(a) Indicate the study’s design with a commonly used term in the title or the abstract	1	-
		(b) Provide in the abstract an informative and balanced summary of what was done and what was found	3	-
Introduction				
Background/rationale	2	Explain the scientific background and rationale for the investigation being reported	4	-
Objectives	3	State specific objectives, including any prespecified hypotheses	4	-
Methods				
Study design	4	Present key elements of study design early in the paper	4, 5	See “Project Design”.
Setting	5	Describe the setting, locations, and relevant dates, including periods of recruitment, exposure, follow-up, and data collection	5, 6	-
Participants	6	(a) Cohort study - Give the eligibility criteria, and the sources and methods of selection of participants. Describe methods of follow-up. Case-control study - Give the eligibility criteria, and the sources and methods of case ascertainment and control selection. Give the rationale for the choice of cases and controls. Cross-sectional study - Give the eligibility criteria, and the sources and methods of selection of participants	5, 6	See “Study Procedures” and “Patient Eligibility Criteria”.
		(b) Cohort study - For matched studies, give matching criteria and number of exposed and unexposed. Case-control study - For matched studies, give matching criteria and the number of controls per case		-
Variables	7	Clearly define all outcomes, exposures, predictors, potential confounders, and effect modifiers. Give diagnostic criteria, if applicable	6	See “Feasibility Outcomes” and “Neuropsychological Outcomes”.
Data sources/measurement	8*	For each variable of interest, give sources of data and details of methods of assessment (measurement). Describe comparability of assessment methods if there is more than one group	5-7	See “Demographic and Clinical Data” to “Data Analysis”.
Bias	9	Describe any efforts to address potential sources of bias	4, 15	Assessment bias is addressed on Page 4; response bias is addressed on Page 15.
Study size	10	Explain how the study size was arrived at	7, 13	Sample size is addressed on Page 13.
Quantitative variables	11	Explain how quantitative variables were handled in the analyses. If applicable, describe which groupings were chosen and why	5-7	See “Demographic and Clinical Data” to “Data Analysis”.
Statistical methods	12	(a) Describe all statistical methods, including those used to control for confounding	7	-
		(b) Describe any methods used to examine subgroups and interactions	7	-
		(c) Explain how missing data were addressed	Figure 1	See Figure 1: Recruitment flow diagram.
		(d) Cohort study - If applicable, explain how loss to follow-up was addressed Case-control study - If applicable, explain how matching of cases and controls was addressed Cross-sectional study - If applicable, describe analytical methods taking account of sampling strategy	15	Strategies to improve participant retention are discussed on Page 15.
		(e) Describe any sensitivity analyses		N/A
Results				
Participants	13*	(a) Report numbers of individuals at each stage of study - e.g., numbers potentially eligible, examined for eligibility, confirmed eligible, included in the study, completing follow-up, and analysed	8	See “Feasibility Outcomes” and Figure 1: Recruitment flow diagram.
		(b) Give reasons for non-participation at each stage	8, 15	-
		(c) Consider use of a flow diagram	8	See Figure 1: Recruitment flow diagram.
Descriptive data	14*	(a) Give characteristics of study participants (e.g., demographic, clinical, social) and information on exposures and potential confounders	7-8	See Tables 1 and 2.
		(b) Indicate number of participants with missing data for each variable of interest	8	See “Feasibility Outcomes” and Figure 1: Recruitment flow diagram.
		(c) Cohort study - Summarize follow-up time (e.g., average and total amount)	8	See “Feasibility Outcomes”.
Outcome data	15*	Cohort study - Report numbers of outcome events or summary measures over time	8-12	See Figure 1 and Table 3.
		Case-control study - Report numbers in each exposure category, or summary measures of exposure	-	-
		Cross-sectional study - Report numbers of outcome events or summary measures	-	-
Main results	16	(a) Give unadjusted estimates and, if applicable, confounder-adjusted estimates and their precision (e.g., 95% confidence interval). Make clear which confounders were adjusted for and why they were included	7-12	-
		(b) Report category boundaries when continuous variables were categorized	21-24	IQR is reported where continuous variables are presented. See Tables 1-3.
		(c) If relevant, consider translating estimates of relative risk into absolute risk for a meaningful time period		N/A
Other analyses	17	Report other analyses done - e.g., analyses of subgroups and interactions, and sensitivity analyses	7	See “Data Analysis”.
Discussion				
Key results	18	Summarize key results with reference to study objectives	12-14	-
Limitations	19	Discuss limitations of the study, taking into account sources of potential bias or imprecision. Discuss both direction and magnitude of any potential bias	15	-
Interpretation	20	Give a cautious overall interpretation of results considering objectives, limitations, multiplicity of analyses, results from similar studies, and other relevant evidence	15	-
Generalizability	21	Discuss the generalizability (external validity) of the study results	13	-
Other information				
Funding	22	Give the source of funding and the role of the funders for the present study and, if applicable, for the original study on which the present article is based	1	See “Funding Statement”.
-		*Give information separately for cases and controls in case-control studies and, if applicable, for exposed and unexposed groups in cohort and cross-sectional studies.	-	-
-		Note: An Explanation and Elaboration article discusses each checklist item and gives methodological background and published examples of transparent reporting. The STROBE checklist is best used in conjunction with this article (freely available on the websites of PLoS Medicine at http://www.plosmedicine.org/, Annals of Internal Medicine at http://www.annals.org/, and Epidemiology athttp://www.epidem.com/). Information on the STROBE Initiative is available at www.strobe-statement.org.	-	-

Appendix 2

**Table 5:** Demographic and Clinical Variables Collected

Outcomes	Data collected
Demographic and Clinical History (obtained from patient records and telephone conversation)	▪ Age ▪ Gender ▪ Highest level of education ▪ Marital status ▪ Current living arrangement ▪ Pre- and post-admit employment status and occupation ▪ Pre- and post-admit functional status ▪ Substance use (smoking, vaping, alcohol, street drugs) ▪ Pre-admission comorbidities
Personal Health Information (obtained from telephone conversation using the C-19 YRS)	▪ Whether or not they sought further medical care after discharge ▪ Physical symptoms experienced relating to: o Breathlessness o Laryngeal issues o Trouble swallowing/eating o Mobility and fatigue o Incontinence o Pain ▪ Psychological symptoms: o Depression o Anxiety o Post-traumatic stress ▪ Cognitive symptoms relating to: o Concentration o Short-term memory o Communication with others ▪ Changes to daily life (personal care and usual activities) ▪ Overall perception of their health status
Clinical Data (recorded from clinical chart and electronic medical chart)	▪ Delirium as assessed by the Intensive Care Delirium Screening Checklist (ICDSC) tool (recorded by nursing staff for clinical purposes) ▪ Other acute neurological sequelae (i.e., clinically diagnosed seizures, clinically diagnosed encephalopathy] ▪ Pharmacological therapies administered and cumulative dosages o Any corticosteroid therapy o Any sedative medications o Any benzodiazepines ▪ Physiological parameters including: o Hypoxemia o Hypercapnia o Hypotension ▪ Major clinical events in hospital (i.e., cardiac arrest) ▪ Highest level of respiratory support received in hospital (i.e., invasive mechanical ventilation, non-invasive mechanical ventilation, high flow nasal cannula, oxygen therapy only, no oxygen therapy required] ▪ Duration of mechanical ventilation, if applicable ▪ Duration of vasopressor therapy, if applicable ▪ Length of ICU stay ▪ Length of hospital stay
Neuroimaging (recorded from electronic medical chart)	▪ Computerized tomography (CT) head, if done for clinical purposes ▪ Magnetic resonance imaging (MRI) brain, if done for clinical purposes ▪ Electroencephalogram (EEG) findings, if done for clinical purposes

Appendix 3

**Table 6:** Psychological Instrument Scoring

Instrument	Response category scale	Scoring
Personal Health Questionnaire (PHQ-9)	Not at all = 0; Several days = 1; More than half the days = 2; Nearly every day = 3	Minimal depression 0-4; Mild depression 5-9; Moderate depression 10-14; Moderately severe depression 15-19; Severe depression 20-27
General Anxiety Disorder (GAD-7)	Not at all = 0; Several days = 1; More than half the days = 2; Nearly every day = 3	Minimal anxiety 0-4; Mild anxiety 5-9; Moderate anxiety 10-14; Severe anxiety 15-21
Impact of Event Scale (IES-R)	Not at all = 0; A little bit = 1; Moderately = 2; Quite a bit = 3; Extremely = 4	Intrusion subscale: The mean response of items 1, 2, 3, 6, 9, 14, 16, 20. Scores can range from 0 to 4. Avoidance subscale: The mean response of items 5, 7, 8, 11, 12, 13, 17, 22. Scores can range from 0 to 4. Hyperarousal subscale: The mean response of items 4, 10, 15, 18, 19, 21. Scores can range from 0 to 4.

Appendix 4

**Table 7:** COVID-19 Yorkshire Rehabilitation Screening (C19-YRS) Telephone Questionnaire

Domain/item	Six months (n = 15)	12 months (n = 10)
Symptoms		
Breathlessness, median (IQR), range		
At rest		
Now	2 (1-5)	0 (0-0)
Pre-COVID	0 (0-1)	0 (0-0)
On dressing yourself		
Now	3 (1-6.5)	0 (0-0)
Pre-COVID	0 (0-1.5)	0 (0-0)
On walking up a flight of stairs		
Now	5 (5-8.5)	3.5 (0.3-6.5)
Pre-COVID	3 (1-4)	0 (0-3.3)
Laryngeal/airway complications		
Changes in sensitivity of throat (e.g., troublesome cough, noisy breathing)		
Yes	7	4
No	8	6
Significance of impact of laryngeal/airway complications 0-10, median (IQR), range	4 (2.5-6.5)	5 (3.8-6.3)
Changes in voice		
Yes	8	6
No	7	4
Significance of impact of voice change (0-10), median (IQR), range	5 (4-5.5)	4 (1.3-4)
Swallowing problem		
Yes	3	4
No	12	6
Significance of impact of swallowing problems (0-10), median (IQR), range	6 (6-7)	2 (2-6)
Nutrition: Weight loss or nutritional concerns		
Yes	3	2
No	12	8
Appetite or interest in eating (0-10), median (IQR), range	0 (0-2.5)	4 (2-6)
Mobility: Problems with walking about (0-10), median (IQR), range		
Now	4 (2.5-6)	1 (0-6)
Pre-COVID	0 (0-1)	0 (0-0.8)
Fatigue: Becoming fatigued more easily		
Yes	12	3
No	3	7
Severity of impact of fatigue on mobility, personal care, activities, or enjoyment of life (0-10), median (IQR), range		
Now	6 (2-8.3)	7 (6.5-7.5)
Pre-COVID	1 (0-3.5)	0 (0-1)
Personal care: Severity of problems with personal care (0-10), median (IQR), range		
Now	1 (0-3)	0 (0-0)
Pre-COVID	0 (0-0)	0 (0-0)
Continence		
New problems with controlling bowel		
Yes	2	2
No	13	8
New problems controlling bladder		
Yes	5	3
No	10	7
Usual activities: Severity of problems in doing usual activities (0-10), median (IQR), range		
Now	4 (0.5-7.5)	4 (1.3-6.8)
Pre-COVID	0 (0-0.5)	0 (0-0)
Pain/discomfort: Severity (0-10), median (IQR), range		
Now	3 (0.5-6)	4 (0.3-6.5)
Pre-COVID	0 (0-1.5)	0 (0-0)
Cognition		
New or worsened difficulty with concentrating		
Yes	8	7
No	7	3
New or worsening difficulties with short-term memory		
Yes	9	8
No	6	2
Cognitive-communication: Change in the way you communicate with people		
Yes	4	7
No	11	3
Significance of impact (0-10), median (IQR), range	3 (1.8-5.3)	6 (5-6.5)
Anxiety: Severity of anxiety symptoms (0-10), median (IQR), range		
Now	3 (1-6.5)	0 (0-3.8)
Pre-COVID	1 (0-2.5)	0 (0-0)
Depression: Severity of depression (0-10), median (IQR), range		
Now	0 (0-3.5)	3.5 (0-4.8)
Pre-COVID	0 (0-0)	0 (0-1.5)
PTSD screen		
Unwanted memories of your illness or hospital admission while awake		
Yes	3	4
No	12	6
Mild	1	2
Moderate	1	2
Severe	1	0
Extreme	0	0
Unpleasant dreams about your illness or hospital admission		
Yes	5	2
No	10	8
Mild	2	1
Moderate	2	1
Severe	1	0
Extreme	0	0
Try to avoid thoughts or feelings about your illness or hospital admission		
Yes	7	6
No	8	4
Mild	2	1
Moderate	4	5
Severe	1	0
Extreme	0	0
Current thoughts about harming self in any way		
Yes	0	0
No	15	10
Global perceived health (0-10), median (IQR), range		
Now	7 (6-8)	7 (5-8)
Pre-COVID	8 (8-9)	9 (8-9)

Appendix 5

**Table 8:** Patients’ Average Z-Scores on the Creyos-6 Tests

Cognitive test	Mean Z-score (SD)	Statistics
Odd one out	-0.18 (1.27)	t(27) = 0.75, p = 0.4598
Rotations	-0.96 (0.97)	t(27) = 5.24, p < 0.0001
Monkey ladder	-0.39 (0.84)	t(27) = 2.46, p = 0.0207
Paired associates	-0.45 (1.00)	t(27) = 2.38, p = 0.0246
Digit span	-1.01 (0.98)	t(27) = 5.45, p < 0.0001
Grammatical reasoning	-1.37 (1.13)	t(27) = 6.42, p < 0.0001
Cognitive domain	Mean Z-score (SD)	Statistics
Verbal processing skills	-1.29 (1.03)	t(27) = 6.63, p < 0.0001
Reasoning ability	-0.59 (0.97)	t(27) = 3.22, p = 0.0033
Short-term memory	-0.41 (1.02)	t(27) = 2.13, p = 0.0427

Appendix 6

**Table 9:** Comparison of Patients Who Received Invasive Mechanical Ventilation and Those Who Did Not on the Creyos-6 Battery Cognitive Domains

Cognitive domain	Invasively ventilated (N = 12)	Not invasively ventilated (N = 16)
	Mean Z-score (SD)	Mean Z-score (SD)
Verbal processing skills	-1.32 (1.25)	-1.33 (1.09)
Reasoning ability	-0.69 (1.34)	-0.32 (1.02)
Short-term memory	-0.30 (0.92)	-0.23 (0.93)

## References

[1] Maresca G, Latella D, Carnazza L, Corallo F, Formica C (2022). Neuropsychological effects of COVID-19: a review. Brain Behav.

[2] Wild CJ, Norton L, Menon DK, Ripsman DA, Swartz RH, Owen AM (2022). Disentangling the cognitive, physical, and mental health sequelae of COVID-19. Cell Rep Med.

[3] Ganesh A, Rosentreter RE, Chen Y (2023). Patient-reported outcomes of neurologic and neuropsychiatric symptoms in mild COVID-19: a prospective cohort study. CMAJ Open.

[4] Sankaranarayanan CS, Lakshmi KR, Senthilnathan M, Poorvika AG, Kumar SS (2025). Cognitive impairment in post-COVID-19 patients: a prospective neuropsychological evaluation study. J Pharm Bioallied Sci.

[5] Ishiyama H, Ishii J, Yoshimura H (2021). Neurological manifestations and long-term sequelae in hospitalized patients with COVID-19. Intern Med.

[6] Elboraay T, Ebada MA, Elsayed M (2025). Long-term neurological and cognitive impact of COVID-19: a systematic review and meta-analysis in over 4 million patients. BMC Neurol.

[7] Stavem K, Einvik G, Tholin B, Ghanima W, Hessen E, Lundqvist C (2022). Cognitive function in non-hospitalized patients 8-13 months after acute COVID-19 infection: a cohort study in Norway. PLoS One.

[8] Schild AK, Goereci Y, Scharfenberg D (2023). Multidomain cognitive impairment in non-hospitalized patients with the post-COVID-19 syndrome: results from a prospective monocentric cohort. J Neurol.

[9] Honarmand K, Malik S, Wild C, Gonzalez-Lara LE, McIntyre CW, Owen AM, Slessarev M (2019). Feasibility of a web-based neurocognitive battery for assessing cognitive function in critical illness survivors. PLoS One.

[10] Balit N, Sun S, Zhang Y, Sharp M (2024). Online unsupervised performance-based cognitive testing: a feasible and reliable approach to scalable cognitive phenotyping of Parkinson's patients. Parkinsonism Relat Disord.

[11] Franco-Rocha OY, Mahaffey ML, Matsui W, Kesler SR (2023). Remote assessment of cognitive dysfunction in hematologic malignancies using web-based neuropsychological testing. Cancer Med.

[12] (2026). Creyos | Digital Cognitive Assessment Platform for Healthcare. https://creyos.com.

[13] Hampshire A, Highfield RR, Parkin BL, Owen AM (2012). Fractionating human intelligence. Neuron.

[14] Kroenke K, Spitzer RL, Williams JB (2001). The PHQ-9: validity of a brief depression severity measure. J Gen Intern Med.

[15] Spitzer RL, Kroenke K, Williams JB, Löwe B (2006). A brief measure for assessing generalized anxiety disorder: the GAD-7. Arch Intern Med.

[16] Weiss DS (2007). The impact of event scale: revised. Cross-Cultural Assessment of Psychological Trauma and PTSD. International and Cultural Psychology Series.

[17] Sivan M, Halpin S, Gee J (2020). Assessing long-term rehabilitation needs in COVID-19 survivors using a telephone screening tool (C19-YRS tool). Adv Clin Neurosci Rehabil.

[18] Yang T, Yan MZ, Li X, Lau EH (2022). Sequelae of COVID-19 among previously hospitalized patients up to 1 year after discharge: a systematic review and meta-analysis. Infection.

[19] Bonazza F, Luridiana Battistini C, Fior G (2022). Recovering from COVID-19: psychological sequelae and post-traumatic growth six months after discharge. Eur J Psychotraumatol.

[20] Spada MS, Biffi AM, Belotti L (2022). Psychological impact of COVID-19 after hospital discharge: a follow-up study on Italian recovered patients. J Affect Disord.

[21] Irisson-Mora I, Salgado-Cordero AM, Reyes-Varón E, Cataneo-Piña DJ, Fernández-Sánchez M, Buendía-Roldán I, Salazar-Lezama MA (2022). Comparison between the persistence of post COVID-19 symptoms on critical patients requiring invasive mechanical ventilation and non-critical patients. PLoS One.

[22] Myhren H, Ekeberg O, Tøien K, Karlsson S, Stokland O (2010). Posttraumatic stress, anxiety and depression symptoms in patients during the first year post intensive care unit discharge. Crit Care.

[23] Honarmand K, Lalli RS, Priestap F, Chen JL, McIntyre CW, Owen AM, Slessarev M (2020). Natural history of cognitive impairment in critical illness survivors. A systematic review. Am J Respir Crit Care Med.

[24] Pandharipande PP, Girard TD, Jackson JC (2013). Long-term cognitive impairment after critical illness. N Engl J Med.

[25] Geocadin RG, Koenig MA, Jia X, Stevens RD, Peberdy MA (2008). Management of brain injury after resuscitation from cardiac arrest. Neurol Clin.

[26] Slutsky AS, Villar J (2019). Early paralytic agents for ARDS? Yes, no, and sometimes. N Engl J Med.

[27] Wood MD, Maslove DM, Muscedere J, Scott SH, Boyd JG (2018). Robotic technology provides objective and quantifiable metrics of neurocognitive functioning in survivors of critical illness:a feasibility study. J Crit Care.

[28] Larsen LK, Møller K, Petersen M, Egerod I (2020). Cognitive function and health-related quality of life 1 year after acute brain injury: an observational study. Acta Anaesthesiol Scand.

[29] Abdelazeem B, Abbas KS, Amin MA, El-Shahat NA, Malik B, Kalantary A, Eltobgy M (2022). The effectiveness of incentives for research participation: a systematic review and meta-analysis of randomized controlled trials. PLoS One.

[30] Edwards PJ, Roberts I, Clarke MJ, DiGuiseppi C, Woolf B, Perkins C (2023). Methods to increase response to postal and electronic questionnaires. Cochrane Database Syst Rev.

